# Antiulcer Activity and Potential Mechanism of Action of the Leaves of *Spondias mombin* L.

**DOI:** 10.1155/2018/1731459

**Published:** 2018-04-26

**Authors:** Samara Alves Brito, Cynthia Layse Ferreira de Almeida, Temístocles Italo de Santana, Alisson Rodrigo da Silva Oliveira, Jéssica Carla Bezerra do Nascimento Figueiredo, Isis Torres Souza, Lécio Leone de Almeida, Márcia Vanusa da Silva, Augusto Santos Borges, Jonathan Wagner de Medeiros, Jacinto da Costa Silva Neto, Rita de Cássia Ribeiro Gonçalves, Rodrigo Rezende Kitagawa, Antônio Euzébio Goulart Sant'Ana, Larissa Araújo Rolim, Irwin Rose Alencar de Menezes, Teresinha Gonçalves da Silva, Germana Freire Rocha Caldas, Almir Gonçalves Wanderley

**Affiliations:** ^1^Department of Pharmaceutical Sciences, Universidade Federal de Pernambuco, Recife, PE, Brazil; ^2^Institute of Chemistry and Biotechnology, Universidade Federal de Alagoas, Maceió, AL, Brazil; ^3^Department of Biological Sciences, Universidade Regional do Cariri, Crato, CE, Brazil; ^4^Department of Biochemistry, Universidade Federal de Pernambuco, Recife, PE, Brazil; ^5^Department of Pharmaceutical Sciences, Universidade Federal do Espírito Santo (UFES), Vitória, ES, Brazil; ^6^Department of Cellular and Applied Molecular Biology, Universidade Estadual de Pernambuco, Recife, PE, Brazil; ^7^Department of Histology and Embryology, Universidade Federal de Pernambuco, Recife, PE, Brazil; ^8^Department of Chemistry, Universidade Federal de Alagoas, Maceió, AL, Brazil; ^9^Analytical Center of Drugs, Medicines and Food, Universidade Federal do Vale do São Francisco, Petrolina, PE, Brazil; ^10^Department of Biological Chemistry, Universidade Regional do Cariri, Crato, CE, Brazil; ^11^Department of Antibiotics, Universidade Federal de Pernambuco, Recife, PE, Brazil; ^12^Graduate Program of Health Sciences, Biologic and Health Sciences Center, Universidade Federal do Maranhão, São Luís, MA, Brazil; ^13^Department of Physiology and Pharmacology, Universidade Federal de Pernambuco, Recife, PE, Brazil

## Abstract

*Spondias mombin* L. is used in folk medicine for the treatment of inflammation and gastrointestinal diseases. Our study investigated the antiulcer activity of *S. mombin* ethanolic extract (SmEE) and its majority compounds gallic acid (GA) and ellagic acid (EA). Phytochemical characterization was performed by HPLC. The SmEE was screened for in vitro antioxidant activities using phosphomolybdenum, ABTS, DPPH, and FRAP assays. The antiulcer activity of SmEE, GA, EA, or GA + EA was evaluated by gastric lesion models induced by absolute ethanol and indomethacin. Following this, it is capable of stimulating mucus production, antisecretory capacity, and the influence of −SH groups and NO in the effect of SmEE. Its healing activity was demonstrated by acetic acid-induced chronic ulcer model. Anti-*Helicobacter pylori* activity was assessed by determining the MIC of the SmEE (64–1024 *μ*g/mL). The HPLC results identified the presence of gallic acid and ellagic acid in SmEE. The extract showed antioxidant activity in vitro. SmEE (50, 100, and 200 mg/kg) reduced the area of ulcerative lesions induced by ethanol in 23.8, 90.3, and 90.2%, respectively. In NSAID model, the SmEE induced protection of 36.8, 49.4, and 49.9%, respectively. GA (10 mg/kg) or EA (7 mg/kg) or the association of GA + EA (10 + 7 mg/kg) inhibited the ethanol-induced lesions in 71.8, 70.9, and 94.9%, respectively, indicating synergistic action. SmEE (100 mg/kg) decreased acid secretion and H^+^ concentration in the gastric contents, increased levels of mucus, and showed to be dependent of −SH groups and NO on the protection of the gastric mucosa. In chronic ulcer model, SmEE reduced the gastric area lesion. SmEE showed anti-*H. pylori* activity. In conclusion, our study showed that SmEE has antiulcerogenic activity. GA and EA are isolated gastric protectors and, when associated, acted synergistically to protect the gastric mucosa.

## 1. Introduction

Peptic ulcer is a public health problem with high rate of morbidity and substantial mortality and has become the focus of experimental and clinical investigations, mainly due to its high prevalence in the global population [1]. Peptic ulcers are usually aggravated by an imbalance between destructive and defensive factors in the stomach [2].

The endogenous destructive factors in the stomach are HCl, pepsin, biliary reflux, lipid peroxidation, and the formation of reactive oxygen species (ROS) and the exogenous factors are excessive use of ethanol, indiscriminate use of nonsteroidal anti-inflammatory drugs (NSAID), stress, smoking, and infection by *Helicobacter pylori* bacteria [3–6]. The defensive factors are mucus-bicarbonate barrier, mucin secretion, surface phospholipids, prostaglandins (PGs), nitric oxide (NO), mucosal blood flow, cell renewal, growth factors, and antioxidant enzymes [2, 4, 5].

Oxidative stress, present in the process of gastric ulceration, increases the formation of ROS that can disrupt epithelial cell integrity. An excess production of ROS metabolites may overwhelm the endogenous antioxidants [7]. In addition, ROS accumulates neutrophils in the tissues of the mucosa during gastric ulceration. Studies have shown that proinflammatory cytokines induce the activation of neutrophils and are strong contributors to the of ulcer damage [8, 9].

Effective therapies for peptic ulcers use alternatives that control acidic hypersecretion and its direct effects on the gastric mucosa. The two main classes of drugs used to treat acid-related disorders include proton pump inhibitors (PPI) that inhibit the hydrogen pump in the parietal cell directly, independently of any membrane receptor stimulation, and histamine type 2 receptor antagonists (H2RAs), which block the histamine receptor on parietal cells thereby reducing hydrogen ion release [10]. PPI is among the most prescribed drugs in the world; however, it may lead to the development of parietal cell hyperplasia of the gastric glands [11]. Long-term use of H2RAs is associated with the development of undesirable effects such as gynecomastia and galactorrhea as well as alteration of the bacterial flora of the gastrointestinal tract [12].

*Spondias mombin* L. of the Anacardiaceae family is native in Brazil [13]. Mainly found in the north and northeast regions [14] and with a growing demand for its fruits and processed products (pulp, juice, and ice cream), it has aroused interest in the agribusiness and grower sectors for commercial operations [15]. The leaves are used in ethnomedicine for the treatment of several topical and systemic inflammatory diseases and for gastrointestinal disorders [16]. Certain pharmacological properties, including anti-inflammatory and antioxidant activities, have been attributed to *S. mombin* [13, 16]. These activities may be to the content of phenolic compound present in *S. mombin* [17, 18].

Phytochemical studies of different parts of *S. mombin* were performed; through HPLC, the ellagic acid was identified as the major compound of the hydromethanolic extract of the leaves [19]. Fraction of the ethyl acetate yielded seven subfractions, out of which the pure gallic acid, coumaroyl, quercetin, and derivatives were obtained [17]. The volatile composition of the leaves was made possible to identify the presence of 41 compounds; the principal component was *β*-caryophyllene and followed by *γ*-cadinene [20]. Besides, the phytochemical screening of the leaves and barks revealed the presence alkaloids, glycosides, saponin, lipid and oil, tannins, flavonoids, terpenoids, and acids [21].

The phytochemicals are defined as chemicals produced by plants, which may exert a beneficial effect on health by different actions such as antioxidant, anti-inflammatory, and modulating gene expression. Indeed, the intake of antioxidants and related bioactive compounds from fruits and vegetables can provide protection against oxidative stress [22–24].

Considering the use of this species in ethnomedicine, its anti-inflammatory and antioxidant activities, our study investigated the antiulcerogenic activity of the *Spondias mombin* ethanolic extract (SmEE), isolated gallic acid (GA), and ellagic acid (EA) and in association between gallic acid and ellagic acid. The potential mechanisms of action of SmEE were also observed.

## 2. Materials and Methods

### 2.1. Reagents and Chemicals

All the reagents below were of analytical grade, and the stock solutions and buffers were prepared with Milli-Q water (Millipore, Billerica, MA, USA). Ethanol, DPPH (2,2-diphenyl-1-picrylhydrazyl), ABTS (2,2′-azino-bis(3-ethylbenzothiazoline-6-sulfonic acid) diammonium salt), TPTZ (2,4,6-tris (2-pyridyl)-s-triazine), tris(hydroxymethyl)aminomethane, sodium chloride, ascorbic acid, Griess reagent, lansoprazole, ranitidine hydrochloride, 2-thiobarbituric acid, EDTA (ethylene diamine tetraacetic acid), N-acetylcysteine, quercetin, gallic acid, ellagic acid, tannic acid and catechin, IU urease (jack bean urease, type III), Alcian blue, N-ethylmaleimide, N*_ω_*-nitro-L-arginine methyl ester, ranitidine, carbenoxolone were obtained from Sigma-Aldrich (St. Louis, MO, USA). Trolox is obtained from Merck, Düsseldorf, Germany; TNF alpha ELISA kit is from eBioscience, San Diego, CA, USA. Historesin is obtained from Leica Biosystems, Wetzlar, Germany, and acetic acid and glucose were obtained from Vetec, Duque de Caxias, Rio de Janeiro, Brazil. Ethyl ether, formaldehyde, and phenolphthalein are from FMaia, Cotia, São Paulo, Brazil; xylazine and ketamine are obtained from Vetbrands, Paulínia, São Paulo, Brazil. Ki-67 protein (code: sc-23900) and BrdU protein (code: sc-32323) are from Santa Cruz Biotechnology, Santa Cruz, CA, USA.

### 2.2. Plant Material and Preparation of Extract

*Spondias mombin* leaves were collected in Cajazeirinhas Municipality, Paraíba, Brazil (S 6°58.34.558^″^–W 37°48.21.424^″^), in July 2014. A voucher specimen was deposited in the Herbarium of the Agronomic Institute (IPA) on the registration number 89987. The leaves (2000 g) were air dried (oven with forced air circulation at 40°C).

Thereafter, the plant material (2000 g) was subjected to cold exhaustive maceration with three separate solvents (2000 mL) of different polarities (hexane, ethyl acetate, and ethanol) under agitation for three consecutive periods of 72 h intervals each. The extracts were filtered, and the solvents were completely removed with the aid of a low-pressure rotary evaporator. The ethanolic extract presented a satisfactory yield (7.3% *w/v*), which was superior to the other extracts.

### 2.3. Phytochemical Study

#### 2.3.1. Thin Layer Chromatography (TLC) Method

The presence of secondary metabolite groups in the SmEE was assessed using TLC, and the following specific chemical developers were used: flavonoids, cinnamic derivatives, phenylpropanoid glycosides (NEU reagent), triterpenes and steroids, mono- and sesquiterpenes (*β*-sitosterol and thymol), coumarins and quinones (KOH), alkaloids (Dragendorff reagent), saponins (vanillin/sulfuric acid reagent), condensed proanthocyanidins, and leucoanthocyanidin (vanillin/HCl reagent) [25–27].

#### 2.3.2. High-Performance Liquid Chromatography (HPLC) Method

The analytical chromatography was performed with ultrapure solvents. In the mobile phase, Milli-Q water was used (Millipore, Billerica, MA, USA). The samples were analyzed by liquid chromatography, equipped with bombs LC-10ADvp, controller SCL-10Avp, degasser DGU-20A_3_, detector SPD-M20A, and auto injector SIL-20A HT (Shimadzu, Kyoto, Japan). Chromatographic separation was performed using a Column C18 (5 *μ*m particle size and 150 × 4.6 mm i.d. (Shimadzu, Kyoto, Japan). The solvent system used contained (A) Milli-Q water and (B) MeOH, with elution gradient flow of 1 mL/min and inject volume of 20 *μ*L, 5%–100% B 0–15 min, 100% B 15–20 min, and 5% B 25 min.

Before injecting in the chromatograph, the SmEE was filtered in through syringe filter Millipore Millex-HV with 0.45 *λ*m of polyvinylidene fluoride (PVDF). The standards used were ellagic acid, tannic acid, gallic acid, and catechin. Standards and extracts were prepared at a concentration of 1 mg/mL. The samples were compared to retention time, UV spectrum, and the coelution *λ* = 260 nm.

The mobile phase was composed of two solvents: solvent A (0.01% trifluoroacetic acid solution diluted in ultrapurified water) and solvent B (100% acetonitrile), following the gradient described in Table 1, with a flow rate of 0.8 mL/min^−1^. The stationary phase was a C^18^ column (Agilent, CA, USA) with dimensions of 250 × 4.6 mm, particle size 5 *μ*m, maintained at 30°C. For the triplicate assay, 40 *μ*L of the sample was injected, which was monitored at wavelengths of 340 and 270 nm. Standards analyzed are as follows: cafeic acid, chlorogenic acid, gallic acid, p-coumaric acid, tannic acid, apigenin, chrysin, fisetin, hesperidin, myricetin, narigenin, resveratrol, rutin, scopoletin, vintexin, harman, and quercetin.

### 2.4. *In Vitro* Antioxidant Activity

#### 2.4.1. Phosphomolybdenum Method

The antioxidant activity of the SmEE was determined by the phosphomolybdenum method based on spectrophotometric determination of the reduction of Mo^4+^ to Mo^5+^, with the subsequent formation of Mo^5+^ phosphate, which has a maximum absorption at 695 nm [28]. The total antioxidant activity (TAA) was expressed relative to ascorbic acid and calculated by using the following formula: % TAA = (*A*_s_ − *A*_c_) × 100/(*A*_a_ − *A*_c_), where *A*_s_ is the absorbance in the presence of the extract, *A*_c_ is the control absorbance (white: without extract), and *A*_a_ is the absorbance of the ascorbic acid.

#### 2.4.2. ABTS: 2,2′-Azino-bis(3-ethylbenzthiazoline-6-sulphonic Acid) Assay

For this test, the methodology described by Re et al. [29] was used. Initially, the radical ABTS^•+^ was formed from the reaction of 7 mM ABTS stock solution with 140 mM potassium persulfate. All experiments were carried out in triplicate. The percentages of oxidative inhibition were calculated and plotted as a function of the reference antioxidant concentration (Trolox) and expressed as Trolox equivalent antioxidant capacity (TEAC) in the unit (*μ*M).

#### 2.4.3. Estimation of 2,2-Diphenyl-1-picrylhydrazyl (DPPH) Radical Scavenging Activity

Free radical sequestering activity was measured via hydrogen donation using the stable radical DPPH [30]. The percentage of inhibition (*I*%) was calculated using the following equation: *I*% = [(Abs_0_ − Abs_1_)/Abs_0_] × 100, where Abs_0_ is the absorbance of the control, and Abs_1_ is the absorbance in the presence of the test compound.

#### 2.4.4. Ferric Reducing Antioxidant Power (FRAP) Assay

This assay was performed according to the method described by Rufino et al. [31], which is based on the reduction of ferric tripyridyltriazine complex to its dark blue ferrous form, in the absence and presence of antioxidants. The results are expressed as Trolox equivalent antioxidant capacity (TEAC) values, calculated with respect to the original FRAP in mmoL Trolox/g.

### 2.5. Animals

Swiss mice (30–35 g) and Wistar rats (180–250 g) of both sexes, obtained from the Department of Physiology and Pharmacology at the Federal University of Pernambuco, were used in experiments. The animals had free access to standard food and water and were kept in separate rooms at 22 ± 2°C with 55–65% humidity with a 12 h light/dark cycle. The animals were maintained in cages with raised wide mesh floors to prevent coprophagy. In all protocols, the animals were euthanized in a CO_2_ chamber (by inhalation). The experimental protocols were submitted to and approved by the Animal Experimentation Ethics Committee of the Federal University of Pernambuco (license number 013615/2015-11) in accordance with the National Institutes of Health's (Washington, DC, 2011) *Guide for the Care and Use of Laboratory Animals*.

### 2.6. Acute Toxicity Study in Mice

An acute toxicity study was performed with Swiss female mice, as described by OECD 420 [32] with slight modifications. The animals were randomly divided into two groups (*n* = 5) and fasted overnight with free access to water. The control group received 0.9% NaCl solution (10 mL/kg), and the treated group received a single dose of 2000 mg/kg SmEE by oral route. The occurrence of death and behavior parameters, signs, and symptoms were observed for 30, 60, 120, 180, and 240 minutes after oral administration as well as daily for 14 days. Food and water consumption and body weight were registered for 14 days. At the end of this period, the number of survivors was recorded to determine the LD_50_.

At the end of the 14 days of observation, all animals were fasted for 12 h with free access to water and, after this, anaesthetized with ketamine (6 mg/kg) and xylazine (60 mg/kg) by intraperitoneal route for blood collection. Blood samples were obtained by cardiac puncture using a syringe and collected in two tubes, one tube containing anticoagulant ethylenediaminetetraacetic acid (EDTA) and one tube without additional anticoagulant, for hematological and biochemical analyses, respectively. After that, the animals were euthanized for necropsy and evaluation of vital organs [33].

### 2.7. Antiulcer Activity

#### 2.7.1. Ethanol-Induced Ulcer

After 16 h of fasting, the rats (*n* = 5–7) were pretreated orally with 0.9% NaCl solution (injured control), lansoprazole (30 mg/kg), SmEE (50, 100, and 200 mg/kg), gallic acid (GA, 10 mg/kg), ellagic acid (EA, 7 mg/kg), or gallic acid + ellagic acid (GA + EA, 10 + 7 mg/kg). The dose values of GA and EA were selected based on the results of the chromatographic study. After 60 mins, all groups were administered 4 mL/kg of absolute ethanol (99.8%) by oral route to induce gastric ulcer according to the method described by Morimoto et al. [34] with slight modifications. After 1 h, the animals were euthanized, the stomachs were photographed, and the lesions were measured by ImageJ software (Bethesda, MD, USA). The results were expressed as the total area of ulcerative lesions (mm^2^). The stomachs pretreated with SmEE were also used to quantify the antioxidant activity of the gastric mucosa (lipid peroxidation and nonprotein sulfhydryl groups). The stomachs pretreated with SmEE, GA, EA, and GA + EA were used for histological analyses.

#### 2.7.2. Determination of Lipid Peroxidation and Nonprotein Sulfhydryl Group Levels

The lipid peroxidation index was determined using the method described by Ohkawa et al. [35]. The tissue was weighed and homogenized in cold KCl (0.15 mol/L) solution for analysis. The absorbance was measured at 532 nm, and the results were expressed as nmol of MDA/mg of protein which reacts with thiobarbituric acid. The levels of nonprotein sulfhydryl groups (−SH groups) in the gastric mucosa were determined using the method described by Sedlak and Lindsay [36]. For this, the tissue was weighed and homogenized in cold EDTA solution (0.02 mol/L). The results were compared with a standard curve using N-acetylcysteine and corrected for the protein content of the initial homogenates, and the concentrations of −SH groups were expressed in nmol of −SH groups/mg of protein.

#### 2.7.3. Histopathology Study

For the histopathologic analyses, gastric mucosa samples were preserved in 10% buffered formaldehyde. Right after, these were washed in buffer, dehydrated in increasing alcohol concentrations in series, and included in historesin glycol methacrylate (Historesin, Leica, Wetzlar, Germany). The 4 *μ*m sections were obtained in a microtome, model RM 2245 (Leica, Wetzlar, Germany) and subject to staining techniques by hematoxylin and eosin (H&E). The slides in duplicate/animal were examined under a light microscope and photographed using Leica camera, model EC3, attached to the microscope and the Leica Application Suite software EZ (Leica, Wetzlar, Germany) used for histopathology [37].

### 2.8. Effect of SmEE on TNF and NO in the Gastric Tissue

Mice were fasted 16 h prior to being pretreated orally with 0.9% NaCl solution (injured control), lansoprazole (30 mg/kg), or SmEE (50, 100, and 200 mg/kg). After 50 mins, all groups (*n* = 5–7) received (by gavage) 0.2 mL of 0.3 M HCl and 60% ethanol solution (HCl/ethanol) for gastric ulcer induction according to the method by Mizui and Doteuchi [38] with slight modifications. The animals were euthanized 1 h after the administration of the ulcerogenic agent, and the stomachs were removed. The gastric tissue of the animals were homogenized in 10% (*w/v*) ice-cold 50 mM phosphate buffer (pH 7.4) and then centrifuged at 11,000 ×g for 10 minutes (4°C) for quantification of tumor necrosis factor-*α* (TNF-*α*) and nitric oxide (NO) levels.

The TNF-*α* concentration was determined using the supernatant of the homogenate by ELISA kit according to the manufacturer's instructions (eBioscience, San Diego, CA, USA), and the values were expressed as pg of cytokines/mL of tissue. The nitrite concentration in the gastric tissue sample was used as an index of nitric oxide production by the Griess reaction [39]. The absorbance from the samples was read in a microplate reader in a spectrophotometer at 560 nm, and the nitrite concentration was determined by comparing the sample absorbance to a standard curve for sodium nitrite. The results were performed in triplicate and expressed in *μ*mol/g of tissue.

### 2.9. Indomethacin-Induced Gastric Ulcer

Wistar rats (*n* = 5–7/group), after 18 h of fasting, were orally pretreated with 0.9% NaCl solution (injured control), ranitidine 60 mg/kg (an H_2_, antihistamine), or SmEE (50, 100, and 200 mg/kg). After 30 minutes after the pretreatment, indomethacin (30 mg/kg) was administered subcutaneously to induce gastric lesions, according to the methodology described by Djahanguiri [40], with modifications. Six hours after the administration of indomethacin, the animals were euthanized, and the stomachs were removed for the determination of gastric lesions, as previously described.

### 2.10. Evaluation of Mucosal Protective Factors

#### 2.10.1. Determination of Mucus Adhering to Gastric Mucus

The experiment was performed according to the Rafatullah et al. [41] method with modifications. After 18 h of fasting, the animals (*n* = 5–7) received by oral route 0.9% NaCl solution, 10 mL/kg (injured control), carbenoxolone (200 mg/kg), or SmEE (100 mg/kg). After 1 hour, under anesthesia (xylazine, 6 mg/kg associated to ketamine, 60 mg/kg, intraperitoneally), the animals were submitted to longitudinal incision for the pylorus ligature. After 4 hours, the animals were euthanized; the glandular portion of the stomach was separated, weighed, and immersed in 10 mL of the 0.1% Alcian blue solution (0.16 M sucrose/0.05 M sodium acetate, pH 5.8). After 2 h of immersion, excess dye was removed in two successive rinses with 7 mL of 0.25 M sucrose, 15 min and 45 min. Each stomach was sequentially transferred to 10 mL of 0.5 M MgCl_2_ solution for 2 h. Four milliliters of dye solution was then shaken vigorously with an equal volume of ether. The resulting emulsion was centrifuged at 176 ×g for 10 min, and the absorbance of the aqueous layer was measured at 595 nm. The amount of blue dye extracted per gram of wet glandular tissue was then calculated. The result was expressed as mg of Alcian blue/g of tissue.

#### 2.10.2. Determination of Gastric Acid Secretion

The experiment was carried out using the pyloric ligature method described by Shay [42], with slight modifications. The animals were divided into four groups (*n* = 5–8): (1) injured control, (2) ranitidine, (3) SmEE, and (4) noninjured—received no treatment. They fasted for 18 h with free access to 5% glucose solution. For pyloric ligature, the animals were anaesthetized (xylazine, 6 mg/kg associated to ketamine, 60 mg/kg, intraperitoneally) and were submitted to longitudinal incision for the pylorus ligature. Immediately after the ligature, they received an intraduodenal 0.9% NaCl solution (injured control, 0.1 mL/100 g), ranitidine (60 mg/kg), or SmEE (100 mg/kg). The abdominal wall was sutured, and four hours after pylorus ligation, the animals were euthanized. The gastric secretion was collected and centrifuged at 176 ×g for 30 min. The gastric content (g), pH values, and the total acidity (mEquiv.[H^+^]/g/4 h) were available.

#### 2.10.3. Involvement of Nitric Oxide (NO) and Sulfhydryl Compounds (−SH Groups) in Gastroprotection

Rats fasted for 18 h were distributed into 11 groups (*n* = 6-7). Four groups received 0.9% NaCl solution (intraperitoneally): four groups L-NAME (N*ω*-nitro-L-arginine methyl ester, 70 mg/kg, intraperitoneally), an inhibitor of the NO-synthase enzyme; three groups NEM (N-ethylmaleimide, 10 mg/kg, intraperitoneally), a sulfhydryl compound blocker [43] to investigate the influence of endogenous NO and −SH groups on the antiulcer effect of SmEE. 30 min after the administration, a 0.9% NaCl solution (injured control), carbenoxolone (100 mg/kg), SmEE (100 mg/kg), or L-arginine (100 mg/kg only for the blocked and not blocked with L-NAME groups) was administered by oral route. One hour later, all the animals received absolute ethanol (4 mL/kg) by oral route to induce gastric lesions. One hour after the administration of ethanol, the animals were euthanized with CO_2_ gas. The stomachs were removed and photographed, and the lesions were counted by computerized planimetry using an ImageJ software (Bethesda, MD, USA). The results were expressed as total area of ulcerative lesions (mm^2^).

### 2.11. Evaluation of Healing Properties of SmEE

#### 2.11.1. Acetic Acid-Induced Gastric Ulcer

Chronic ulcer induction was based on the study of Takagi et al. [44] with some modifications. The animals were divided into three groups (*n* = 7), fasted for 16 h, and after, the animals were anaesthetized (xylazine, 6 mg/kg associated to ketamine, 60 mg/kg, intraperitoneally) for the surgical exposure of the stomach, and 0.05 mL of 30% acetic acid was injected into the subserosal layer of the external wall of the stomach. One day after the surgery, daily treatment began, and the animals were treated orally once a day for 14 consecutive days with 0.9% NaCl solution (injured control), ranitidine (60 mg/kg), or SmEE (100 mg/kg). During the treatment, the animals were observed for signs of toxicity, such as piloerection, diarrhea, changes in locomotor activity, or mortality, and the body weight was recorded. On the 15th day, the rats were euthanized, the stomachs were removed and photographed, and the injuries were measured by ImageJ software (Bethesda, MD, USA). The results were expressed as the total area of ulcerative lesions (mm^2^).

#### 2.11.2. Histological Analyses

The stomachs with chronic ulcers were sectioned and set in 10% buffered formalin. After setting, the samples were washed with water, immersed in 70% ethyl alcohol for 3-4 days, and embedded in paraffin. 5 *μ*m thick paraffin sections were taken and stained with hematoxylin/eosin (H&E) and periodic acid-Schiff (PAS). Histological analysis of the gastric sections was carried out using an automated microscopy system MICRO DIP.

#### 2.11.3. Immunohistochemical Analysis

The immunohistochemical for Ki-67 and bromodeoxyuridine (BrdU) was performed in 4 *μ*m thick sections in paraffin of samples containing representative portions of the ulcerated area. Initially, the samples were deparaffinized in xylene and hydrated [45].

The expression of Ki-67 and BrdU proteins was detected using the free-biotin method in conjugation with HRP (horseradish peroxidase). The antigenic retrieval was performed by using a pressure cooker for two minutes. The slides were cooled to room temperature, and endogenous peroxidase was blocked by using BSA (bovine serum albumin) for 1 h. After cooling, the slides were incubated overnight with primary monoclonal anti-mouse antibody for Ki-67 protein (code: sc-23900, dilution 1 : 200) and for BrdU protein, IIB5 (code: sc-32323, dilution 1 : 200) (Santa Cruz Biotechnology, Santa Cruz, CA, USA). Then, a HRP visualization system was used. After washing, slides were incubated with diaminobenzidine chromogen solution (DAB), washed in water, counterstained with hematoxylin, dehydrated, and mounted. Cells immunoreactive for Ki-67 and BrdU were detected by the presence of a dark reddish-brown chromogen in the nucleus or nucleus/cytoplasm, respectively, on epithelial cells of the lesion area. The reactivity was indicated using the following scores: positive mild reactivity (in 10–15% of the analyzed cells), moderate reactivity (in 25–50% of the analyzed cells), strong reactivity (in above 50% of the analyzed cells), or negative (in less than 10% analyzed cells).

### 2.12. Evaluation of Anti-*Helicobacter pylori* Activity

Anti-*Helicobacter pylori* (ATCC 43504) activity was assessed by determining the minimum inhibitory concentration (MIC) of the SmEE (64–1024 *μ*g/mL) using the spectrophotometric method of broth microdilution according to the guidelines of the Clinical and Laboratory Standards Institute (CLSI) [46]. Microplate wells were filled with 100 *μ*L of liquid growth medium (brain-heart infusion supplemented with 10% fetal calf serum) containing different concentrations of SmEE. The absorbance was read in a spectrophotometer (*λ* = 620 nm), and the microplates were then incubated at 36-37°C in the presence of 10% CO_2_ for 72 h. The test was performed in triplicate and repeated at least three times, together with growth controls (absence of sample) and control group (culture medium containing different concentrations of test agent to control for color). The MIC was determined graphically and was defined as the lowest concentration of antibacterial agent at which there was a sharp decline (90%) in absorbance. Amoxicillin (0.065–4 *μ*g/mL) was used as the standard.

#### 2.12.1. Evaluation of Inhibitory Activity against Urease

Urease inhibitory activity was determined by quantifying the ammonia produced via the urease-catalyzed hydrolysis of urea, according with the method described by Tanaka et al. [47]. 25 *μ*L of 4 IU urease (jack bean urease, type III (Sigma-Aldrich, St. Louis, MO, USA) and 25 *μ*L of the samples at different concentrations were added to a 96-well microplate and incubated for 2 h at room temperature. Boric acid was used as a standard. Percentage inhibition was calculated with the equation (1 − ABS sample/ABS control) × 100, where ABS is the absorbance. The half-maximal effective concentration (EC_50_) of substrate hydrolysis was calculated based on the inhibitory effect of several SmEE concentrations.

#### 2.12.2. Inhibition of NO Production

The murine macrophage line RAW 264.7 (ATCC TIB-71) (adherent cells) at 1 × 10^6^ cells/well/mL in DMEM supplemented with FBS (DMEM-10) was incubated with SmEE and LPS (5 *μ*g/mL) for 24 h at 37°C in 5% CO_2_ atmosphere for this assay. NO production was determined by measuring the accumulation of nitrite, a stable end-product, in the culture supernatant according to the Griess reaction. The absorbance was read at 540 nm, and the nitrite concentration in the medium was calculated using sodium nitrite as a standard. Cells incubated with LPS only were used as a positive control. The assays were followed by growth control and macrophage viability after the experiments by MTT-tetrazolium assay [48].

#### 2.12.3. Nitric Oxide (NO) Scavenging Assay

The assay was based on the methodology presented by Marcocci et al. [49], with adaptations. Initially, we performed the preparation of SNP (1.25 mM) in phosphate buffer, pH 7.0 (0.1 M). After obtaining this solution, 50 *μ*L of it was incubated with 50 *μ*L of SmEE at different concentrations (25, 50, 100, 200, 400, and 800 *μ*g/mL) for 1 hr at room temperature. After the incubation, 100 *μ*L of Griess reagent (1% *w/v* sulfanilamide, 0.1% *w/v* naphthylethylenediamine, and 2.5% *v/v* orthophosphoric acid) was added and the reaction mixture was then read at 540 nm in a microplate reader iMark (BioRad, Washington, USA). Trolox was used as standard control (25, 50, 100, 200, 400, and 800 *μ*g/mL).

### 2.13. Statistical Analysis

The results were expressed as mean ± standard error of the mean (SEM). The differences between means were determined by Student's *t*-test for unpaired samples or analysis of variance (ANOVA) followed by Dunnett's multiple comparisons test. Statistical analysis was performed using GraphPad Prism 7.0 (GraphPad Software Inc., La Jolla, CA, USA). The level of significance for rejection of the null hypothesis was set at 5% (*p* < 0.05).

## 3. Results and Discussion

### 3.1. Phytochemical Screening

The phytochemical analysis from the ethanolic extract of the leaves of *S. mombin* (SmEE) revealed the presence of flavonoids, cinnamic derivatives, triterpenoids, steroids, mono- and sesquiterpenes, alkaloids, proanthocyanidins, and leucoanthocyanidins. However, the presence of coumarins and quinones was not observed.

### 3.2. High-Performance Liquid Chromatography (HPLC)

From the chromatographic analysis of SmEE, it was possible to identify two compounds using analytical standards based on the similarity between retention times and ultraviolet absorption spectrum (*λ* = 260 nm). They were identified as gallic acid (101.52 ± 0.60 *μ*g/mL) and ellagic acid (68.74 ± 1.50 *μ*g/mL).

The presence of ellagic acid in the hydromethanolic extract of this species and its antioxidant activity were described by Da Silva et al. [19]. Murakami et al. [50] described a marked inhibitory effect by the extract on acid secretion and the occurrence of stress-induced gastric lesions, and these effects may be attributed to the inhibition of H^+^ and K^+^-ATPase activity.

Gallic acid is described as a gastroprotector, which inhibits mitochondrial apoptosis [51]. Higher concentrations of this bioactive compound in food or leaves along with the presence of ellagic acid in vegetal extracts have been found to increase its antioxidant capacity [51–54].

### 3.3. Phosphomolybdenum

The antioxidant activity of the SmEE determined by the phosphomolybdenum method was 82.80 ± 0.70% compared to that of the ascorbic acid, which was considered to be 100%. The antioxidant activity of SmEE may be related to the higher content of total phenols.

### 3.4. DPPH, ABTS, and FRAP

The EC_50_ value of the SmEE for antioxidant activity was 5.53 ± 0.10 *μ*g/mL, whereas that of the ascorbic acid (positive control) was 4.00 ± 0.10 *μ*g/mL, which is commonly used as a standard for antioxidant activity [30].

The TEAC (antioxidant activity total equivalent of Trolox) per g of extract was 798.13 mM, and the extract used in our study presented a sequestering activity of ABTS radicals of 43.93%. Among the metal reduction-based antioxidant methods, the FRAP method reduces the Fe(III)-TPTZ complex to Fe(II)-TPTZ at a low pH [31]. Using this method, we calculated a value of 2492.67 *μ*M Trolox/g for this extract. The antioxidant properties of the SmEE were also determined as the free radical scavenging ability, using the DPPH and ABTS methods, and reducing power, via the FRAP method. This antioxidant property has been revealed in a chemical study to be due to the *S. mombin* leaf extracts and was attributed to the presence of quercetin, rutin, and ellagic acid [19].

Biologically active antioxidants may help protect the gastric mucosa against cell damage caused by oxidative stress, as well as enhance the defense systems against degenerative diseases [7, 55]. Studies have associated the administration of antioxidants (synthetic or natural) with protection of the gastric mucosa against necrotic agents, including ethanol and nonsteroidal anti-inflammatory drugs [56, 57]. In particular, omeprazole, a proton pump inhibitor, has additional actions that contribute to its antiulcer activity, including its *in vitro* antioxidant capacity and inhibition of DNA fragmentation (antiapoptosis) of gastric mucosal cells [58]. The *in vitro* antioxidant study showed that the ethanolic extract of *S. mombin* contains classes of bioactive compounds involved in antiulcer processes, and this observation may contribute to the justification of its use in traditional medicine.

### 3.5. Acute Toxicity

The administration of SmEE (2000 mg/kg) showed no evidence of toxicity or death in the female mice. The LD_50_ for SmEE was greater than 2000 mg/kg. With respect to food, water consumption, and weight gain, it was found that the extract did not cause significant changes during the 14 days after administration, as shown in Table 2. The corporal mass of the group treated with the extract did not differ from the control group. Macroscopic analysis of the target organs of the animals treated with SmEE did not show significant differences in weight, color, or texture in relation to the control group (data not shown). These parameters are important pathophysiological indicators that may be affected by metabolic reactions caused by toxic substances [59].

The administration of SmEE also did not induce alteration in the hematological and biochemical parameters (Table 3). These data show that *S. mombin* extract does not exert any acute toxic effect.

Uchendu and Isek [60] conducted an acute toxicity study in rats with the hydroalcoholic extract of leaves from *S. mombin* (500, 1000, and 2000 mg/kg) via an intraperitoneal injection and did not find any lethality or other treatment-related symptoms of the extract in all groups.

### 3.6. Ethanol-Induced Ulcers

**Figure 1:**
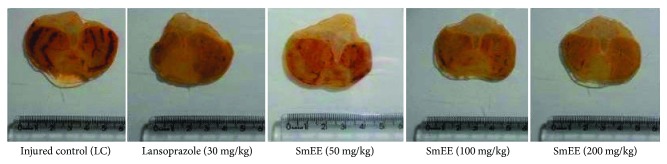
Effect of different doses of ethanolic extract of the leaves of *S. mombin* (SmEE) on the severity of gastric lesion (gross analysis) examined in ethanol-induced gastric ulceration model. These photographs are typical of such tissues.

The administration of ethanol caused extensive damage to the gastric mucosa with hemorrhagic erosions in the injured control group. Oral administration of the SmEE (50, 100, and 200 mg/kg) and lansoprazole (30 mg/kg) significantly inhibited gastric lesions induced by ethanol, in comparison to the injured control (Table 4 and Figure 1), by 23.84, 90.33, 90.27, and 89.26%, respectively.

**Figure 2:**
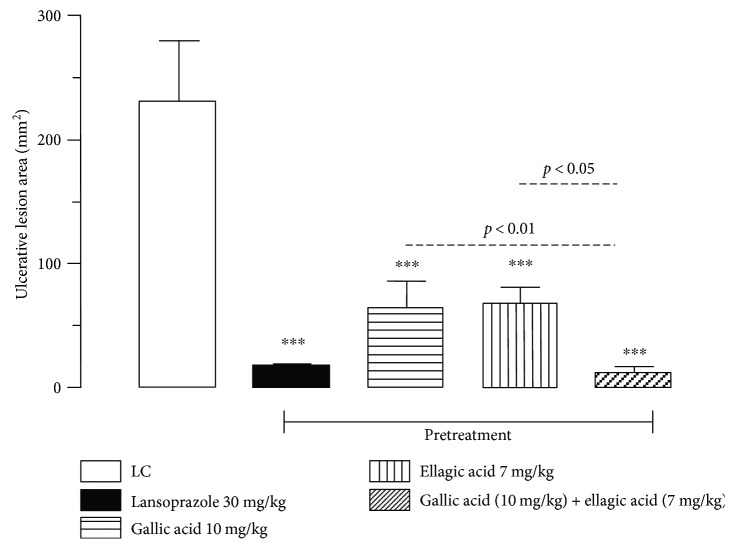
Effect of the oral pretreatment with gallic acid, ellagic acid, and gallic acid + ellagic acid on gastric lesions induced by ethanol in rats. LC: injured control. The results are expressed as the mean ± SEM (*n* = 6/group). One-way analysis of variance (ANOVA), followed by Dunnett's multiple comparisons test; ^∗∗∗^*p* < 0.001. Student's *t*-test for unpaired samples; *p* < 0.05 and *p* < 0.01.

**Figure 3:**
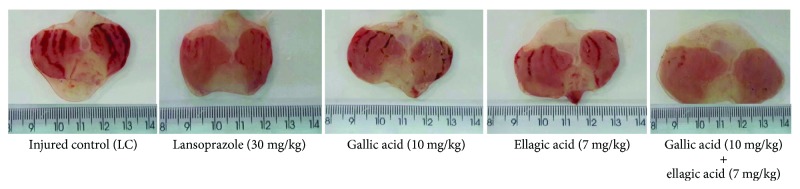
Effect of gallic acid and ellagic acid isolated and in association with the severity of gastric lesion (gross analysis) examined in ethanol-induced gastric ulceration model. These photographs are typical of such tissues.

Oral administration of the gallic acid (10 mg/kg), ellagic acid (7 mg/kg), association (gallic acid (10 mg/kg) + ellagic acid (7 mg/kg)), and lansoprazole (30 mg/kg) significantly inhibited gastric lesions induced by ethanol, in comparison to the injured control group by 71.82, 70.92, 94.96, and 92.82%, respectively (Figures 2 and 3). In addition, a synergistic activity of the combination of these two compounds was observed, with reduction of gastric lesions of 82.12% in relation to gallic acid and of 82.67% in relation to ellagic acid. This is the first study evaluating the gastroprotective activity of gallic acid and ellagic acid.

The ethanol-induced ulcer model is commonly used in animal experiments, and lesions occur predominantly in the glandular portion of the stomach [61]. This necrotizing agent reduces gastric defensive mechanisms, starting from the formation of reactive oxygen species (ROS), including superoxide anions, hydroxyl radicals, and lipid peroxides [62]. Oral administration of ethanol causes linear hemorrhagic lesions, extensive submucosal edema, mucosal friability, inflammatory cell infiltration, and epithelial cell loss in the stomach [63]. These damages to the gastric mucosa may be associated with a decrease in the levels of glutathione, intracellular oxidative stress, changes in membrane permeability, and mitochondrial membrane depolarization, leading to cell death [64].

Oral pretreatment with SmEE (50, 100, and 200 mg/kg) and lansoprazole (30 mg/kg) showed a significant decrease to 62.06, 62.06, 72.41, and 44.82%, respectively, in the rate of lipid peroxidation when compared with the injured control 0.29 ± 0.02 (Table 4).

The level of the sulfhydryl groups (−SH groups) in animals in the ethanol-injured control group was 1.80 ± 0.32 nmol/mg of protein. Oral treatment with SmEE (50, 100, and 200 mg/kg) and lansoprazole (30 mg/kg) prevented a reduction in the−SH groups after the administration of ethanol (Table 4).

Caldas et al. [45] showed that in comparison to the levels observed in noninjured animals, animals subjected to ethanol-induced damage exhibit an increase in the levels of malondialdehyde (MDA), as well as a decrease in the levels of the nonprotein sulfhydryl groups (−SH groups).

Our results showed that for the SmEE (50, 100, and 200 mg/kg) and lansoprazole (30 mg/kg), a significant decrease in lipid peroxidation, as evidenced by reduced levels of malondialdehyde, by 37.93, 37.93, 27.59, and 55.17%, respectively, was observed. The administration of ethanol was accompanied by a reduction in MDA concentration, which may contribute to the protective effects of SmEE. According to Alvarez-Suarez et al. [62], lipid peroxidation is one of the most commonly used indexes in the evaluation of gastroprotection and is possibly related to the ability to scavenged oxygen-free radicals.

### 3.7. Histopathology

#### 3.7.1. Pretreatment with SmEE in Ethanol-Induced Ulcer

**Figure 4:**
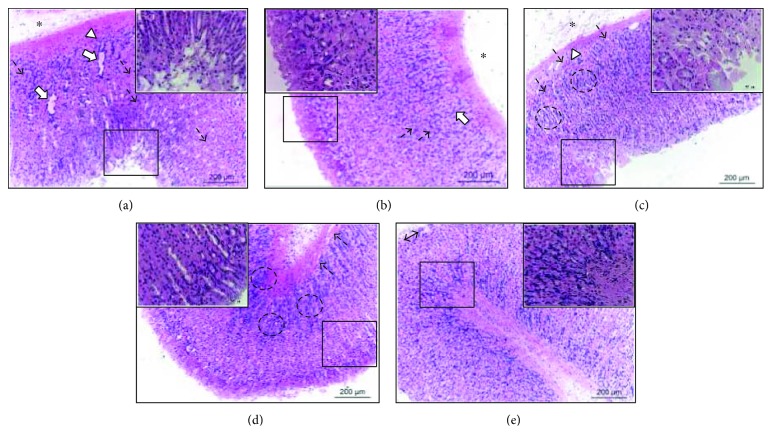
Histopathology of the gastric mucosa of rats of the experimental groups pretreated with 0.9% NaCl (injured control, a), lanzoprazole (30 mg/kg, b), and ethanolic extract of the leaves of *Spondias mombin* (SmEE 50, 100, and 200 mg/kg; c, d, and e, resp.) in ethanol-induced ulcer. Enlarged detail indicates the simple columnar epithelium of the pits. The arrows indicate gastric glands. The dashed arrows indicate congestion of blood capillaries. The asterisk (^∗^) indicates edema of the lamina propria. The arrowhead (∆) indicates mononuclear inflammatory infiltrate diffuse. The dashed circles (◌) indicate gastric glands. Magnification, 100x.

In the histopathological analysis of the stomachs from the injured control group, disorganization of the simple columnar epithelium of the pits and gastric glands, congestion of blood capillaries, edema of the lamina propria, and mononuclear inflammatory infiltrate diffuse were observed (Figure 4(a)). The lansoprazole group showed well-preserved simple columnar epithelium of the pits and gastric glands, despite the moderate presence of diffuse edema of the lamina propria and congestion of the blood capillaries (Figure 4(b)).

Pretreatment with SmEE prevented the occurrence of injury in the surface layers of the gastric mucosa by ethanol.

In the SmEE group (50 mg/kg), it was possible to verify the poorly preserved gastric mucosa-presenting scrubs of the simple columnar epithelium, necrosis of the surface epithelium and gastric glands, congestion of blood capillaries, mononuclear inflammatory infiltrate, and diffuse edema of the lamina propria (Figure 4(c)). However, the gastric mucosa remained well preserved in the groups treated with higher doses but showed the presence of discrete congestion of the blood capillaries in the SmEE group (100 mg/kg) (Figure 4(d)) and discrete areas of exfoliations of the simple columnar epithelium (Figure 4(e)).

#### 3.7.2. Pretreatment with GA, EA, and GA + EA in Ethanol-Induced Ulcer

**Figure 5:**
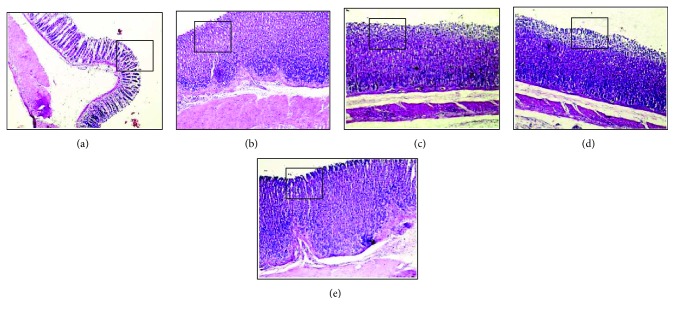
Histopathology of the gastric mucosa of rats of the experimental groups pretreated with 0.9% NaCl (injured control, a), lanzoprazole (30 mg/kg, b), gallic acid (10 mg/kg, c), ellagic acid (7 mg/kg, d), and gallic acid + ellagic acid (10 + 7 mg/kg, e) in ethanol-induced ulcer. The square indicates the simple columnar epithelium. Magnification, 40x.

In the histopathological analysis of the stomachs, the injured control group showed the disorganization of the simple columnar epithelium (Figure 5(a)). The lansoprazole group showed well-preserved simple columnar epithelium (Figure 5(b)).

Pretreatment with GA, EA, or GA + EA prevented the occurrence of injury in the surface layers of the gastric mucosa by ethanol.

In the GA and EA groups, it was possible to verify a moderate diffuse exfoliation in the upper epithelial layer of the gastric mucosa (Figures 5(c) and 5(d)). The association GA + EA group showed well-preserved simple columnar epithelium (Figure 5(e)).

These data are relevant histologically, as the gastric mucosa is one of the most important tissues due to its absorptive and secretory functions [65]. Gastric mucosa exposed to ulcerogenic necrotizing agents such as indomethacin and alcohol or to ischemia develops histopathological characteristics and ultrastructural and functional alterations, which result in injuries [66].

### 3.8. Effect of SmEE on TNF and NO in the Gastric Tissue

The level of tumor necrosis factor-*α* (TNF-*α*) in the gastric tissue was measured by using an enzyme-linked immunosorbent assay kit. The injured control group (2.38 ± 0.08 pg/tissue) showed increased levels of TNF-*α*, as shown in Table 5. The 50, 100, and 200 mg/kg extracts of *S. mombin* significantly reduced the levels of TNF-*α* by 62.61, 54.62, and 72.27%, respectively. In addition, lansoprazole significantly reduced TNF-*α* by 12.93% compared to that after control treatment.

TNF is an important mediator of the acute inflammatory response and is involved in the apoptosis of injured gastric mucosa by various agents, such as other proinflammatory cytokines [67]. Apoptosis is associated with a loss of mucosal integrity subjected to stress, impairment, and microvascular hemorrhage and plays an important role in the development of ulcers [68]. HCl/ethanol administration activates the innate immune system and promotes increased TNF levels in gastric tissue [69].

Nworu et al. [16] investigated the effects of an *S. mombin* methanol extract on inflammation and concluded that the extract can alleviate inflammatory responses, possibly via the suppression of cytokine production, such as TNF-*α*. Our results revealed the ability of SmEE to inhibit the levels of proinflammatory cytokine mediators, thus showing its anti-inflammatory effect on the HCl/ethanol-induced gastric ulcers.

The effect of pretreatment with SmEE and lansoprazole on nitric oxide (NO) levels in the HCl/ethanol-induced gastric lesions is given in Table 5. Animals pretreated with SmEE (50, 100, and 200 mg/kg) showed a significant increase (730.21, 410.43, and 161.51%, resp.) in the level of NO compared with injured control (2.78 ± 0.03 *μ*mol/g of tissue). Similarly, the level of NO was also significantly elevated (160.79%) in the lansoprazole group.

NO increases blood flow, maintains gastric microcirculation, increases mucus secretion in the gastric mucosa, inhibits the activation of leukocytes within the microcirculation, and inhibits the inherent release of reactive oxygen metabolites and proteases [70].

### 3.9. Indomethacin-Induced Gastric Ulcer

**Figure 6:**
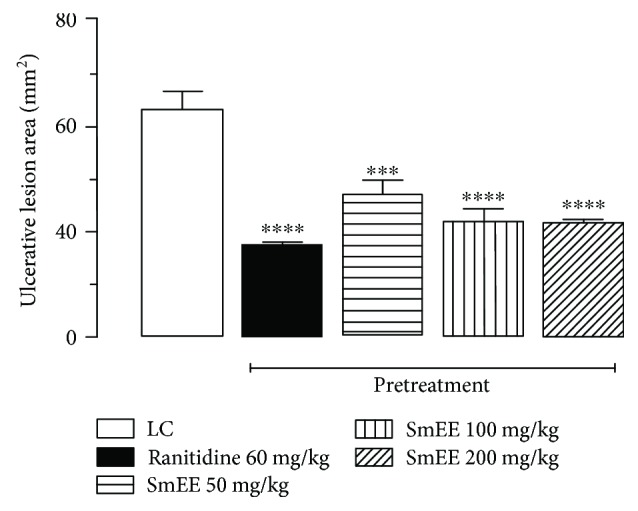
Effect of the oral pretreatment with ethanolic extract of the leaves of *Spondias mombin* (SmEE) on gastric lesions induced by indomethacin in rats. LC: injured control. The results are expressed as the mean ± SEM (*n* = 5–7/group). One-way analysis of variance (ANOVA), followed by Dunnett's multiple comparisons test; ^∗∗∗^*p* < 0.001 and ^∗∗∗∗^*p* < 0.0001.

**Figure 7:**
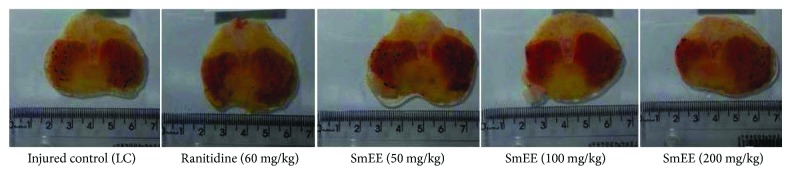
Effect of different doses of ethanolic extract of the leaves of *Spondias mombin* (SmEE) on the severity of gastric lesion (gross analysis) examined in indomethacin-induced gastric ulceration model. These photographs are typical of such tissues.

The subcutaneous administration of indomethacin (30 mg/kg), a nonsteroidal anti-inflammatory drug (NSAID), caused injury to the gastric mucosa of 56.21 ± 4.30 mm^2^ in the injured control. Animals pretreated with SmEE at doses of 50, 100, and 200 mg/kg (per os) showed a significant inhibition of the lesions by 36.8, 49.40, and 49.90%, respectively, in relation to the control group. The animals that received ranitidine (60 mg/kg) presented an inhibition of the lesions corresponding to 59.60% (Figures 6 and 7).

Sabiu et al. [71] have shown antiulcer and antioxidant effects of *Spondias mombin* and *Ficus exasperata* aqueous extracts (100 and 200 mg/kg) in rats treated orally for 21 days (once daily) following indomethacin-induced (30 mg/kg) ulcers.

Gastric ulcer induction by NSAIDs occurs through the inhibition of cyclooxygenases and thus prostaglandin synthesis [66]. In the stomach, prostaglandins play an important protective role by stimulating the secretion of bicarbonate and mucus and the maintenance of mucosal blood flow [72]. Prostaglandins are also responsible for regulating mucosal cell renewal. Thus, the suppression of prostaglandin synthesis by NSAIDs causes increased susceptibility to gastric mucosal lesions [1].

Considering that gallic acid and ellagic acid were identified in SmEE, it is possible to suggest that the antiulcerogenic activity of extract can be associated, at least in part, with the synergistic interaction of these compounds described for SmEE.

### 3.10. Effect of SmEE on the Production of Gastric Mucus

**Figure 8:**
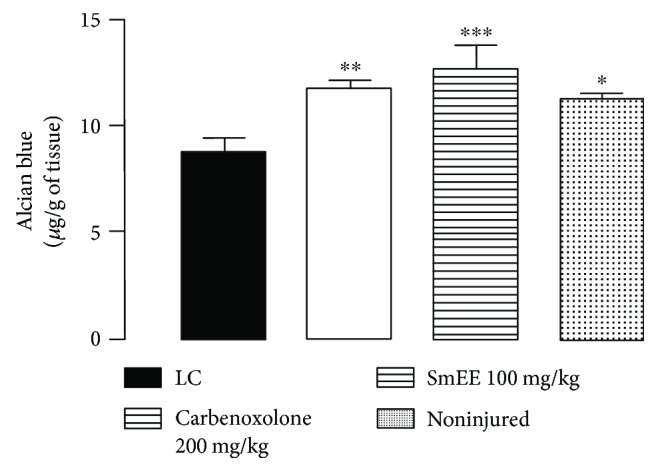
Effect of ethanolic extract of the leaves of *Spondias mombin* (SmEE) on the production of gastric mucus. The results are expressed as the mean ± SEM (*n* = 5–7/group). LC: injured control group. One-way ANOVA, followed Dunnett's multiple comparisons test; ^∗^*p* < 0.05, ^∗∗^*p* < 0.005, and ^∗∗∗^*p* < 0.001.

Ligation of the pylorus-injured control group animals showed a significant decrease in the levels of gastric mucus (8.79 ± 0.67 *μ*g of Alcian blue/g of tissue) compared to those of the false-operated group (11.30 ± 0.23 *μ*g of Alcian blue/g of tissue). Treatment with SmEE (100 mg/kg) and carbenoxolone (200 mg/kg) significantly increased the production of mucus by 44.25 and 33.84%, respectively, compared to that of the injured control group (LC) (Figure 8).

Some studies [73–75] have associated the antiulcer activity of plant materials with stimulation of gastric mucus production as one of the antiulcer mechanisms.

Increased mucus secretion is an important mechanism of gastric mucosal defense against necrotizing agents [76, 77]. In addition, when associated with bicarbonate production, it may play a significant role in the process of inhibiting ulcers to protect newly formed cells from acid and peptic injury [78].

### 3.11. Effect of SmEE on Gastric Acid Secretion

After 4 h of ligation of the pylorus, it was observed that the intraduodenal administration of SmEE (100 mg/kg) and ranitidine (60 mg/kg) reduced the amount of gastric acid secretion by 27.43 and 75.22%, respectively, and the total acidity remaining by 38.80 and 45.78%, respectively. There was an increase in pH in the SmEE and ranitidine groups compared to the pH of the injured control group (Table 6).

Current therapeutic techniques to treat gastric ulcers are based on antisecretory drugs [79], whose adverse effects include the rebound effect of acid hypersecretory secretion [11]. This has contributed to the increasing demand for natural products to treat gastric ulcers with fewer adverse effects.

The *S. mombin* extract decreased acid secretion, as evidenced by an increase in pH and a decrease in H^+^ concentration in the gastric contents. These parameters were evaluated using the pylorus ligature model, an important procedure that shows the possible changes of the parameters related to gastric acid secretion [80]. In this model, the extract was administered intraduodenally, and the data obtained indicated that the *S. mombin* extract presented systemic effects and that the observed effect is not only related to a local neutralization of the gastric content.

### 3.12. Involvement of Nitric Oxide (NO) and Sulfhydryl Compounds (−SH Groups) in Gastroprotection

N*_ω_*-Nitro-L-arginine methyl ester (L-NAME) and N-ethylmaleimide (NEM) exacerbated ethanol-induced gastric lesions by 95.48 and 173.99%, respectively, compared to effects in the groups pretreated with NaCl solution. SmEE (100 mg/kg, p.o.) and carbenoxolone (100 mg/kg, p.o.) significantly inhibited the ulcerative lesions induced by absolute ethanol, both in the absence and presence L-NAME and NEM. There was a significant difference among groups NaCl (i.p.) and SmEE (p.o), L-NAME (i.p.) and SmEE (p.o), and NEM (i.p.) and SmEE (p.o.) (Table 7), suggesting that the antiulcer effect of SmEE depended partially on the production or presence of NO and SH compounds, which are involved in mucosal protection against harmful injuries. Table 7 shows that treatment with L-arginine significantly reverted the deleterious effect of L-NAME on the gastric mucosa.

The SH groups are responsible for increasing the production of and maintaining mucus stability, through the disulfide bridges, and are involved in maintaining gastric integrity, thereby limiting the production of free radicals involved in tissue damage [45, 81]. Their relatively high concentrations indicate possible implications in gastroprotection [24, 45].

Nitric oxide is one of the most important defensive endogenous agents in the gastric mucosa [82]. NO has a dual role in gastroprotection. At high concentrations, it acts as a proinflammatory mediator [16] as observed in not blocked L-arginine. However, at lower concentrations, in blocked L-arginine, NO exerts increased blood flow and maintains the gastric microcirculation. This observation with L-arginine was also verified in a study by [83].

### 3.13. Acetic Acid-Induced Gastric Ulcer

**Figure 9:**
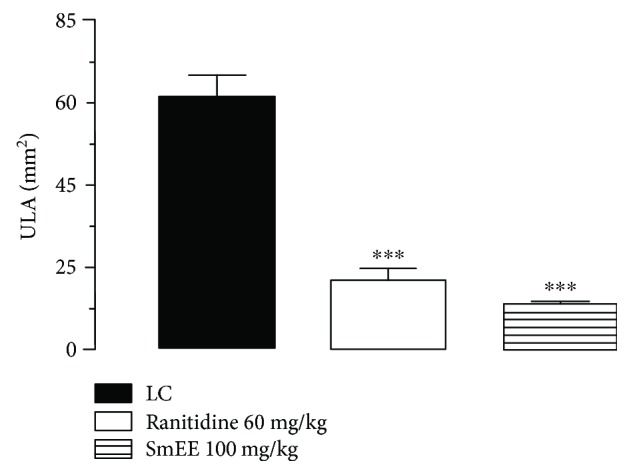
Effect of ethanolic extract of the leaves of *Spondias mombin* (SmEE) on healing of the gastric mucosa in the rats. The results are expressed as the mean ± SEM (*n* = 7/group). One-way ANOVA, followed by Dunnett's multiple comparisons test; ^∗∗∗^*p* < 0.001.

Our results showed that the treatment with SmEE (100 mg/kg) and ranitidine (60 mg/kg) for 14 consecutive days decreased the area of chronic ulcer by 93.48 and 90.30%, in comparison to that of the control group (61.00 ± 1.98 mm^2^), as displayed in Figure 9. Our results also showed that the SmEE sped up the healing of acetic acid-induced chronic ulcers.

This model highly resembles human peptic ulcers in terms of its pathological features, healing process, and cycle of recurrence [80]. We were therefore able to evaluate the healing capacity of drugs [81] and antiulcer phytochemistry materials [45, 84].

Luminal aggressor factors, such as HCl secretion and pepsin, delay ulcer healing. Inhibition of these factors by acid inhibitory drugs (H_2_ blockers and proton pump inhibitors) is one of the major components of current peptic ulcer therapy [85].

During the 14 days of treatment, SmEE or ranitidine did not produce any visible signs of toxicity.

**Figure 10:**
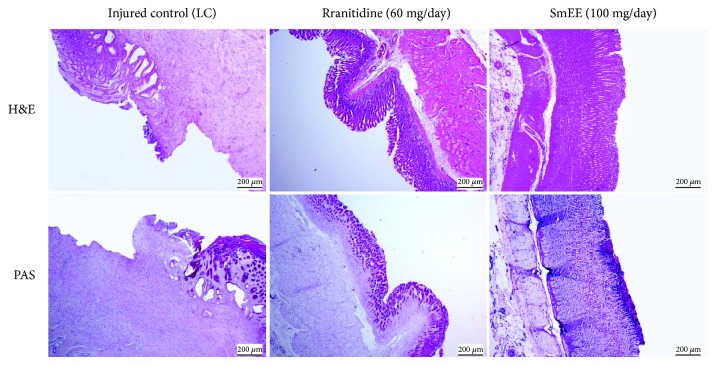
Photomicrographs of gastric mucosa stained with H&E and PAS of the rats subjected to induction of chronic ulcer by 30% acetic acid. Animals were treated orally with 0.9% NaCl solution (LC: injured control), ranitidine (60 mg/kg), or ethanolic extract of the leaves of *Spondias mombin* (SmEE, 100 mg/kg) for 14 days. The filled arrow indicates the absence of the epithelial layer (ulcer area internal), and the dashed arrow indicates epithelial layer remaining (ulcer edge). Hematoxylin/eosin (H&E) and periodic acid-Schiff staining (PAS); magnification, 40x.

In the histological analysis of the stomachs, the H&E-stained sections revealed well-defined ulcers with complete destruction of the mucosal and submucosal layer caused by acetic acid in animals of the control group. The stomachs of rats treated orally with SmEE (100 mg/kg) and ranitidine (60 mg/kg) showed regeneration compared to those of the control group. PAS staining also showed increased mucus production, as viewed through the areas intensely stained pink in the epithelial layer of the mucosa (Figure 10). This demonstrates the involvement of the cells in the release of mucus, which protects the mucosa, as shown by the intense tone of pink after PAS staining.

Gastric mucus is the first barrier in preventing the formation of ulcers [86], and hydrophobicity plays a key role in protecting the gastric membrane against noxious agents in the lumen [87].

**Figure 11:**
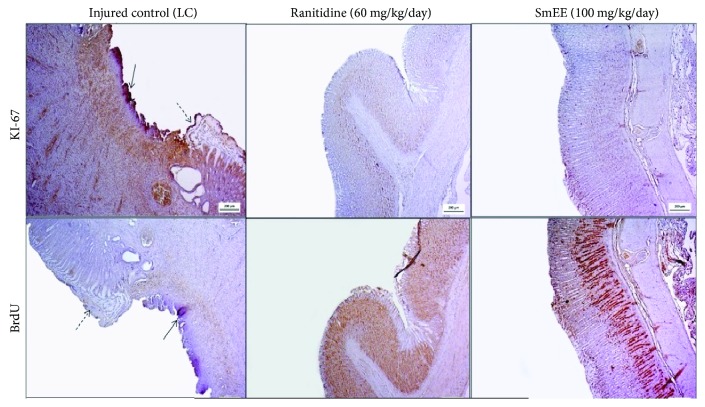
Immunohistochemical analysis for Ki-67 and BrdU of the gastric mucosa of the rats subjected to induction of chronic ulcer by 30% acetic acid. Animals were treated orally with 0.9% NaCl solution (LC, injured control), ranitidine (60 mg/kg), or ethanolic extract of the leaves of *Spondias mombin* (SmEE, 100 mg/kg) for 14 days. The filled arrow indicates the absence of the epithelial layer (ulcer area internal), and the dashed arrow indicates epithelial layer remaining (ulcer edge). Microphotographs depict the immunoreactivity for Ki-67 and BrdU in the groups, magnification 200x.

Immunohistochemical investigation using monoclonal antibodies against Ki-67 and BrdU showed strong reactivity and increased BrdU-positive nuclei and moderate reactivity for Ki-67 in the gastric mucosa of animals treated with SmEE and ranitidine for 14 days, compared to those of the control group, which showed no reactivity for the two markers due to destruction of the epithelial layer, as shown in Figure 11.

The Ki-67 antigen is a nuclear matrix protein that is expressed in proliferating cells during the G1, S, G2, and M phases of the cell cycle, but not in quiescent cells (G0 phase) [87]. BrdU, a synthetic nucleoside of thymidine that is incorporated into DNA during the S phase, is expressed in proliferating cells [88, 89]. Cell proliferation plays an important role in wound healing, and our results suggest that the treatment with SmEE promoted the regeneration of the gastric cells. OECD [32] also observed the 1,8-cineole antiulcerogenic capacity with Ki-67 and BrdU expression in the process of gastric healing.

### 3.14. *Helicobacter pylori*

*H. pylori* plays a role in several gastric diseases. SmEE showed an anti-*H. pylori* activity (MIC = 256 *μ*g/mL) with 90.94 ± 0.68% inhibition. Amoxicillin showed a MIC < 0.5 *μ*g/mL. Current anti-*H. pylori* therapy fails in more than 20% of gastric ulcer disease cases, primarily due to antimicrobial resistance and patient nonadherence [90]. Studies have specifically described the antibiotic activity of herbal products against this bacterium [91, 92].

In a review by Ayala et al. [90], the pharmacological aspects of plant extracts, in addition to their anti-*H. pylori* activity and antisecretory, antioxidant, anti-inflammatory, antihemorrhagic, and gastric ulcer resolution activities, have been described.

*H. pylori* infects half of the world's population and is recognized as the main cause of chronic gastritis and peptic ulcers [93], whereby an infection induces an inflammatory response and changes in the gastric microenvironment. Host immune cells, especially neutrophils and macrophages, release inflammatory mediators and large quantities of reactive oxygen species (ROS) and reactive nitrogen species (RNS), which are related to an increased risk of stomach cancer.

**Figure 12:**
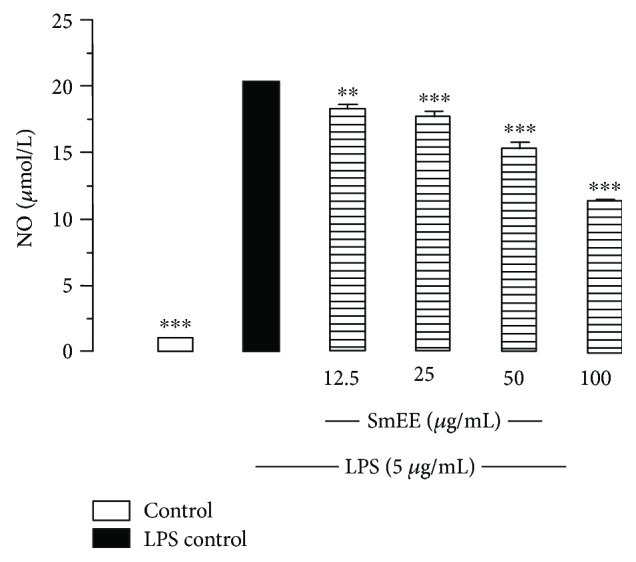
Effect of ethanolic extract of the leaves of *Spondias mombin* (SmEE) on lipopolysaccharide- (LPS-) induce oxide nitric (NO) production in macrophages. Adherent cells (1 × 10^6^) were incubated for 24 h with extract and LPS (5 mg/mL). Cells incubated just with LPS were used as a positive control and cell in culture medium (DMEM) as a control group. Nitrite concentrations in the medium were determined using Griess reagent. The results are expressed as the mean ± SEM for three different experiments performed in triplicate. One-way ANOVA, followed by Dunnett's multiple comparisons test; ^∗∗^*p* < 0.005 and ^∗∗∗^*p* < 0.005 versus LPS control.

The ability of *H. pylori* to avoid immune response leads to persistent local inflammation, which, in turn, results in large amounts of ROS and RNS being produced [94]. The extract was not able to achieve 50% inhibition of urease activity at the tested concentrations. Our results revealed that the SmEE exerted immunosuppressive activity on lipopolysaccharide- (LPS-) stimulated NO generation with a reduction of approximately 44.23% at a concentration of 100 *μ*g/mL (Figure 12).

To study the involvement of *H. pylori* in inflammation, macrophages were stimulated with LPS, which activates macrophage functions and the release of inflammatory mediators, including the generation of NO, and the secretion of proinflammatory cytokines [95]. As most of the NO generated is converted immediately into nitrites, the nitrite level was measured in cell culture supernatants after treatment with SmEE (12.5, 25, 50, and 100 *μ*g/mL) to investigate their potential anti-inflammatory activity, as indicated by the inhibition of the LPS-stimulated NO, implying an anti-inflammatory activity to this extract. The inhibition of the generated NO by the tested extract may be due to a direct scavenging capacity of NO, an inhibition of the inducible nitric oxide synthase (iNOS) pathway or a modulation of other factors in the NO cascade, such as transcriptional factors.

SmEE was able to inhibit the growth of *H. pylori* by 90%. It was also involved in one of the causative mechanisms underlying the pathological process of infection such as increased production of reactive species. *H. pylori* triggers iNOS expression and activity in macrophages, leading to the production of increased amounts of NO, which plays an important role in the immune response of the gastric mucosa by causing local inflammation and injury [96, 97]. Additionally, as NO is mutagenic, its excess production may be linked to *H. pylori* infection-related stomach cancer [96].

To investigate the radical scavenging activity of SmEE, we subjected the extract to an NO scavenging assay, which revealed that the SmEE radical scavenging activity at its maximum (800 *μ*g/mL) concentration was 78.29%. Trolox (800 *μ*g/mL), which was used as a standard control, reduced free radicals by 73.77% (Table 8).

Plants and their biomolecules have been reported to be alternatives to antibiotics against resistant human pathogens due to their proven effectiveness and availability [98, 99] against important mediators of provocative processes that are responsible for chronic diseases, such as ulcers, inflammation, and bacterial infections [100–102]. They act by helping the cell wall and DNA to reduce and neutralize reactive species such as NO generated during normal metabolic processes [97].

## 4. Conclusion

Our study showed that SmEE has antiulcerogenic activity mediated by antioxidant activity, stimulation of the gastric mucus production or involvement of the sulfhydryl groups and nitric oxide, besides antisecretory and anti-*Helicobacter pylori* activities. Together, all these mechanisms can contribute to the chronic ulcer healing promoted by the *Spondias mombin* ethanolic extract. GA and EA are isolated gastric protectors and when associated acted synergistically to protect the mucosa against gastric lesions induced by ethanol.

## Figures and Tables

**Table 1:** Gradient system used in mobile phase analysis by HPLC method.

	Time (min)	Solution A (%)	Solution B (%)
Linear gradient	0–40	90–60	10–40
Isocratic	40–50	60	40
Linear gradient	50–60	60–90	40–10

**Table 2:** Effect of ethanolic extract of the leaves of *Spondias mombin* (SmEE) on food and water consumptions and body weight in female mice for 14 days.

Parameters	Treatment	
	Control (0.9% NaCl solution)	SmEE (2000 mg/kg)
Food consumption (g)	31.36 ± 1.75	31.79 ± 1.37
Water consumption (mL)	51.14 ± 3.46	58.50 ± 3.03
Initial body weight (g)	30.71 ± 0.68	31.43 ± 0.87
End body weight (g)	34.29 ± 0.68	35.83 ± 0.83

Values represent the mean ± SEM (*n* = 5/group). Student's *t*-test for unpaired samples.

**Table 3:** Effect of ethanolic extract of the leaves of *Spondias mombin* (SmEE) on hematological and biochemical parameters in female mice 14 days after treatment.

Parameters	Treatment	
	Control (0.9% NaCl solution)	SmEE (2000 mg/kg)
*Hematological parameters*		
RBC (10^6^/*μ*L)	8.87 ± 0.27	9.32 ± 0.26
Hb (g/dL)	15.15 ± 0.41	14.99 ± 0.34
Ht (%)	43.57 ± 2.24	48.00 ± 1.32
MCV (fL)	50.33 ± 0.95	51.43 ± 0.53
MCH (pg)	17.40 ± 0.87	16.09 ± 0.28
MCHC (g/dL)	32.12 ± 0.17	31.20 ± 0.31
RDW (%)	12.30 ± 0.25	12.76 ± 0.29
Platelets (10^3^/*μ*L)	682.80 ± 49.24	612.00 ± 33.83
*Biochemical parameters*		
Creatinine (mg/dL)	0.18 ± 0.20	0.28 ± 0.20
ALP (mg/dL)	196.60 ± 20.46	236.70 ± 29.04
AST (mg/dL)	164.00 ± 25.29	224.00 ± 31.92
ALT (mg/dL)	64.60 ± 3.71	72.20 ± 11.11
BUN (mg/dL)	53.80 ± 1.57	56.57 ± 7.67

RBC: red blood cell; Hb: hemoglobin; Ht: hematocrit; MCV: mean corpuscular volume; MCH: mean corpuscular hemoglobin; MCHC: mean corpuscular hemoglobin concentration; RDW: red cell distribution width; ALP: alkaline phosphatase; AST: aspartate aminotransferase; ALT: alanine aminotransferase; BUN: blood urea nitrogen. Values represent the mean ± SEM (*n* = 5/group). Student's *t*-test for unpaired samples.

**Table 4:** Effect of the oral pretreatment with ethanolic extract of the leaves of *Spondias mombin* (SmEE) on gastric lesions induced by absolute ethanol in rats.

Pretreatment	Absolute ethanol-induced ulcer		
	Lesion area (mm^2^)	LPO (nmol of MDA/mg protein)	−SH groups (nmol/mg protein)
Injured control (LC)	229.80 ± 11.00	0.29 ± 0.02	1.80 ± 0.32
Lansoprazole (30 mg/kg)	24.67 ± 3.50^∗∗∗^	0.13 ± 0.02^∗∗∗^	4.72 ± 0.56^∗∗^
SmEE (50 mg/kg)	175.00 ± 17.78^∗∗^	0.18 ± 0.00^∗∗∗^	4.66 ± 0.42^∗^
SmEE (100 mg/kg)	22.20 ± 3.22^∗∗∗^	0.18 ± 0.01^∗∗∗^	4.75 ± 0.85^∗∗^
SmEE (200 mg/kg)	22.35 ± 2.14^∗∗∗^	0.21 ± 0.01^∗∗^	4.49 ± 0.36^∗^

LPO: lipid peroxidation; −SH groups: nonprotein sulfhydryl groups. The results are expressed as the mean ± SEM (*n* = 5–7/group). ANOVA followed Dunnett's multiple comparisons test; ^∗^*p* < 0.05, ^∗∗^*p* < 0.01, and ^∗∗∗^*p* < 0.001.

**Table 5:** Effect of the oral pretreatment with ethanolic extract of the leaves of *Spondias mombin* (SmEE) on TNF-*α* and NO production in gastric lesions induced by HCl/ethanol in mice.

Pretreatment	HCl/ethanol-induced ulcer	
	TNF-*α* production (pg/mL of tissue)	NO production (*μ*mol/g of tissue)
Injured control (LC)	2.32 ± 0.04	2.78 ± 0.01
Lansoprazole (30 mg/kg)	2.02 ± 0.02^∗∗∗∗^	4.47 ± 0.00^∗∗∗∗^
SmEE (50 mg/kg)	0.89 ± 0.00^∗∗∗∗^	20.30 ± 0.19^∗∗∗∗^
SmEE (100 mg/kg)	1.08 ± 0.02^∗∗∗∗^	11.41 ± 0.18^∗∗∗∗^
SmEE (200 mg/kg)	0.66 ± 0.02^∗∗∗∗^	4.49 ± 0.08^∗∗∗∗^

The results are expressed as the mean ± SEM (*n* = 5–7/group). ANOVA followed by Dunnett's multiple comparisons test, ^∗∗∗∗^*p* < 0.0001.

**Table 6:** Effect of intraduodenal administration of ethanolic extract of the leaves of *Spondias mombin* (SmEE) on gastric secretion in Wistar rats subjected to pylorus ligature.

Treatment	pH	[H^+^] (mEq/g/4 h)	Gastric content (g)
Noninjured	3.94 ± 0.39^∗∗^	4.43 ± 0.98^∗∗∗^	0.15 ± 0.03^∗∗∗^
Injured control	2.94 ± 0.33	12.19 ± 1.59	1.13 ± 0.19
SmEE (100 mg/kg)	3.50 ± 0.43^∗^	7.46 ± 0.85^∗∗∗^	0.82 ± 0.20^∗∗^
Ranitidine (60 mg/kg)	3.59 ± 0.38^∗^	6.61 ± 1.71^∗∗∗^	0.28 ± 0.06^∗∗∗^

The results are expressed as the mean ± SEM (*n* = 5–8/group). One-way ANOVA, followed by Dunnett's multiple comparisons test; ^∗^*p* < 0.05, ^∗∗^*p* < 0.01, and ^∗∗∗^*p* < 0.0005.

**Table 7:** Effect of oral administration of ethanolic extract of the leaves of *Spondias mombin* (SmEE) on gastric lesions induced by ethanol in Wistar rats pretreated with N*ω*-nitro-L-arginine methyl ester (L-NAME, 70 mg/kg) or N-ethylmaleimide (NEM, 10 mg/kg).

Pretreatment	Treatment (p.o.)	Dose (mg/kg)	Lesion area (mm^2^)	Inhibition (%)
0.9% NaCl (i.p.)	LC	—	56.68 ± 2.83	—
	Carbenoxolone	100	5.40 ± 0.47^∗∗∗^	90.47%
	SmEE	100	32.80 ± 2.38^∗∗∗^	42.13%
	L-Arginine	200	320.30 ± 101.5^∗∗∗^	+465.10%

L-NAME (i.p.)	LC	—	120.80 ± 3.33	—
	Carbenoxolone	100	24.22 ± 1.01^∗∗∗^	79.95%
	SmEE	100	47.00 ± 2.59^∗∗∗^	61.09%
	L-Arginine	200	74.44 ± 12.80^∗∗∗^	38.38%

NEM (i.p.)	LC	—	155.30 ± 9.8	—
	Carbenoxolone	100	44.90 ± 3.15^∗∗∗^	70.51%
	SmEE	100	70.60 ± 3.93^∗∗∗^	54.54%

The results are expressed as the mean ± SEM (*n*=6-7). LC: injured control group. One-way ANOVA, followed by Dunnett's multiple comparisons test; ^∗∗∗^*p* < 0.001.

**Table 8:** Percent inhibition induced by SmEE in the NO^•^ scavenging assay.

Concentration (*μ*g/mL)	Inhibition (%)	
	SmEE	Trolox
25	7.07 ± 2.01	30.20 ± 1.09
50	2.62 ± 3.04	63.98 ± 2.62
100	19.35 ± 6.87	74.22 ± 0.52
200	48.21 ± 4.11	70.70 ± 0.42
400	65.20 ± 2.26	70.19 ± 0.38
800	78.29 ± 1.78	73.77 ± 1.58

The results are expressed as the mean ± SEM for three different experiments performed in triplicate.
